# Optoacoustic biomarkers of lipids, hemorrhage and inflammation in carotid atherosclerosis

**DOI:** 10.3389/fcvm.2023.1210032

**Published:** 2023-11-09

**Authors:** Angelos Karlas, Nikolina-Alexia Fasoula, Michael Kallmayer, Christoph Schäffer, Georgios Angelis, Nikoletta Katsouli, Mario Reidl, Felix Duelmer, Kenana Al Adem, Leontios Hadjileontiadis, Hans-Henning Eckstein, Vasilis Ntziachristos

**Affiliations:** ^1^ Institute of Biological and Medical Imaging, Helmholtz Zentrum München, Neuherberg, Germany; ^2^ Chair of Biological Imaging at the Central Institute for Translational Cancer Research (TranslaTUM), School of Medicine, Technical University of Munich, Munich, Germany; ^3^ Department for Vascular and Endovascular Surgery, Klinikum Rechts der Isar, Technical University of Munich (TUM), Munich, Germany; ^4^ DZHK (German Centre for Cardiovascular Research), partner site Munich Heart Alliance, Munich, Germany; ^5^ Chair for Computer Aided Medical Procedures and Augmented Reality, Department of Informatics, Technical University of Munich, Munich, Germany; ^6^ Department of Biomedical Engineering, Healthcare Engineering Innovation Center (HEIC), Khalifa University, Abu Dhabi, United Arab Emirates; ^7^ Department of Electrical and Computer Engineering, Aristotle University of Thessaloniki, Thessaloniki, Greece

**Keywords:** MSOT, RSOM, stroke, carotid artery disease, vulnerable plaque, unstable plaque, molecular imaging

## Abstract

Imaging plays a critical role in exploring the pathophysiology and enabling the diagnostics and therapy assessment in carotid artery disease. Ultrasonography, computed tomography, magnetic resonance imaging and nuclear medicine techniques have been used to extract of known characteristics of plaque vulnerability, such as inflammation, intraplaque hemorrhage and high lipid content. Despite the plethora of available techniques, there is still a need for new modalities to better characterize the plaque and provide novel biomarkers that might help to detect the vulnerable plaque early enough and before a stroke occurs. Optoacoustics, by providing a multiscale characterization of the morphology and pathophysiology of the plaque could offer such an option. By visualizing endogenous (e.g., hemoglobin, lipids) and exogenous (e.g., injected dyes) chromophores, optoacoustic technologies have shown great capability in imaging lipids, hemoglobin and inflammation in different applications and settings. Herein, we provide an overview of the main optoacoustic systems and scales of detail that enable imaging of carotid plaques *in vitro*, in small animals and humans. Finally, we discuss the limitations of this novel set of techniques while investigating their potential to enable a deeper understanding of carotid plaque pathophysiology and possibly improve the diagnostics in future patients with carotid artery disease.

## Introduction

Carotid artery disease is a major cause of stroke and disability globally. In an attempt to detect early enough the “vulnerable plaque”, or else the plaque that is at high risk to rupture and cause stroke, several imaging modalities have been developed and employed. Main goal of all such modalities is to identify and quantify morphological and pathophysiological features of the plaque tissue which are histologically proven to be associated with rupture and relevant events, such as intraplaque hemorrhage (IPH), thin fibrous cap, ulceration, inflammation and a lipid-rich necrotic core (LRNC) (1).

Ultrasound (US) is the most commonly used imaging technique in carotid atherosclerosis. However, US techniques do not provide direct molecular information and are characterized by low imaging contrast. By sending ultrasound waves in tissues and detecting the waves reflected back to the transducer, US provides structural and functional plaque visualizations, yet without revealing direct information regarding the molecular consistency of the plaque. For example, purely morphological features such as the echogenicity or other textural characteristics of the plaque have been used to identify vulnerable carotid plaques so far (2, 3).

Moreover, computed tomography (CT), and in particular CT angiography (CTA), enable the effective diagnosis of carotid stenoses and is used for preoperative surgical planning or postoperative assessment in vascular patients (4). Nevertheless, even if current CT scanners need only seconds to image the region of interest, employ ionizing radiation and often necessitate the use of possibly toxic contrast agents. Magnetic resonance imaging (MRI) provides detailed visualizations of plaque microanatomy and consistency without the use of ionizing radiation (5). Despite advantages, MRA is characterized by long scanning times and costly/bulky equipment. Moreover, positron emission tomography (PET) is the gold-standard in imaging vascular inflammations or infections (6). PET provides molecular/metabolic information yet with the administration of radionuclides and the use of complex and non-portable systems.

**Figure 1:**
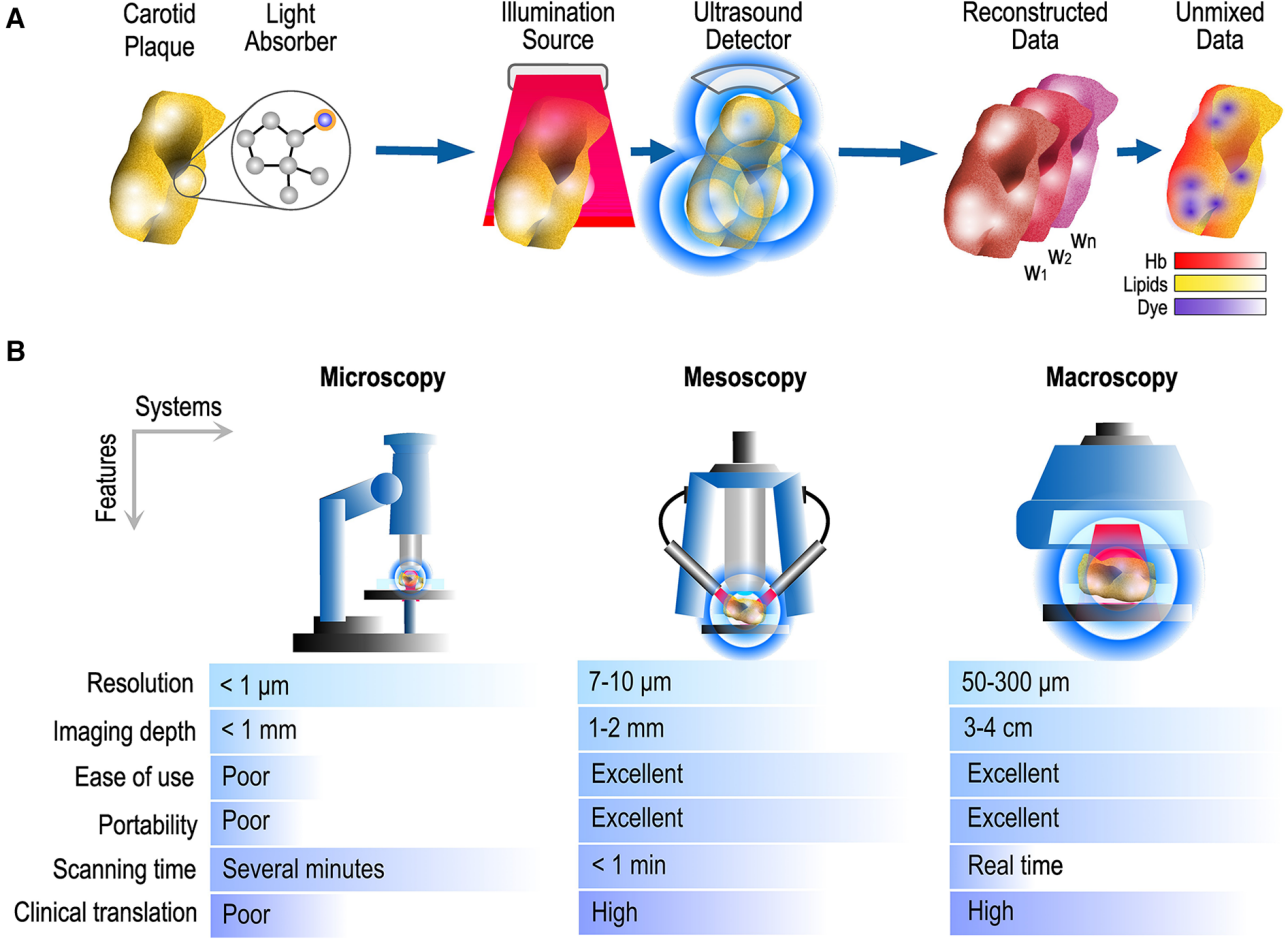
Optoacoustic imaging and usual systems. (**A**) Pipeline for the production of optoacoustic images: After illuminating the tissue/sample with multiple light wavelengths (*w*_1_, *w*_2_, …, *w*_n_), the optoacoustic data can be spectrally unmixed to reveal the spatial distribution of endogenous and injected chromophores. (**B**) Common systems and scales of detail/information provided along with main technical characteristics. Hb, hemoglobin.

Optoacoustics (OA), also termed photoacoustics, describes a group of technologies that provide comprehensive vascular imaging by combining the high portability of US with the advantages of employing non-ionizing radiation and not requiring the administration of contrast agents. By employing laser light at the visible (400–700 nm) or near-infrared (NIR, 700–1,000 nm) range, optoacoustics provides high-resolution imaging of oxygenated (HbO_2_) and deoxygenated (Hb) hemoglobin, lipids and collagen based only on the absorption of light by these tissues (7, 8). In particular, optoacoustic imaging takes advantage of the production of US waves as a response to tissue light absorption to image the above-mentioned molecules and provide insights into different aspects of vascular physiology and pathophysiology with high acoustic resolution and optical contrast (Figure 1A). The hybrid nature of optoacoustics enables the conduction of high-quality imaging at different scales of detail and imaging depths (8). In fact, optoacoustic microscopy (OAM) techniques offer sub-micrometer resolutions at depths up to 300 µm, mesoscopic techniques resolutions of 7–10 µm at depths of 1–2 mm and macroscopic implementations resolutions of few hundreds of micrometers at depths of up to several centimeters (3–4 cm), depending on tissue type and system characteristics (Figure 1B). OA has been recently used not only in *ex vivo* studies of excised carotid plaques but also *in vivo* animal and human studies.

**Figure 2:**
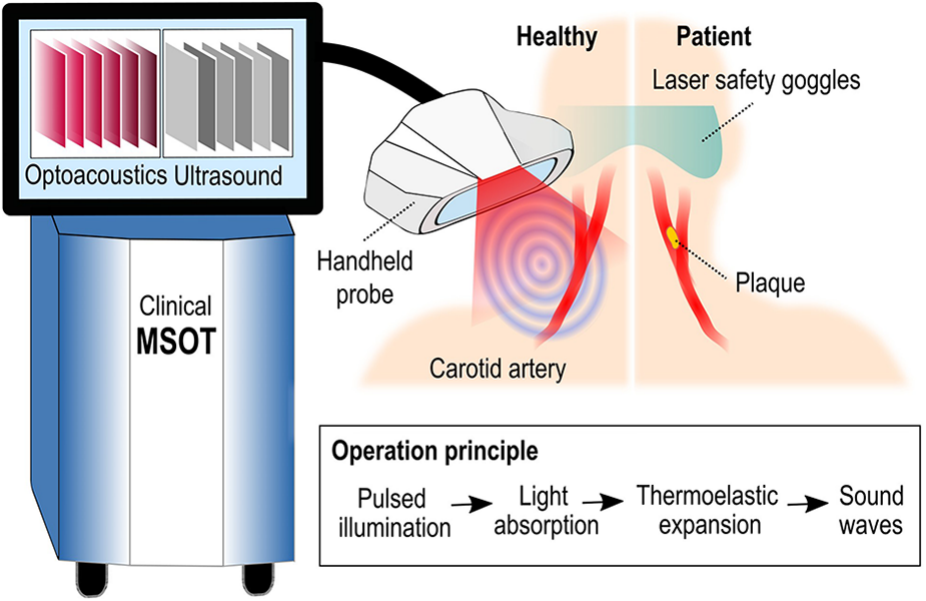
Configuration and principle of operation of clinical MSOT and diagram of imaging the carotid artery.

Multispectral optoacoustic tomography (MSOT), else called photoacoustic tomography (PAT), and raster-scan optoacoustic mesoscopy (RSOM) are the optoacoustic technologies that are the most frequently used in clinical, and in particular, vascular applications. On the one hand, MSOT operates in real-time (25–50 frames/s), reaches depths of 3–4 cm by employing near-infrared light and offers resolutions of 200–300 µm by using US detectors of 4–12 MHz central frequency. By illuminating tissue at several different wavelengths (Figure 2), MSOT allows for the spectral unmixing of the recorded optoacoustic data, or in other words the detection of specific molecular absorbers based on their known absorption spectrum over the wavelength range employed. For example, based on this principle, MSOT can differentiate among oxy- (HbO_2_) and deoxygenated (Hb) hemoglobin, lipids and water within the imaged tissue areas (9–12). MSOT may be further enhanced by the use of externally administrated contrast agents providing multiaspect imaging of different tissue processes. Modern handheld systems are equipped with simultaneous and co-registered US imaging. On the other hand, RSOM needs 45–60 s to record a high-resolution (7–10 µm) volumetric image of the skin (penetration depth of 1–2 mm) using visible green light (532 nm) and US detectors of 50–100 MHz (7). Recent fast-RSOM implementations have decreased the image acquisition times down to one second per frame (13). Both technologies are hand-held and label-free.

The nature of blood vessels as carriers of blood throughout the body renders them an ideal focus for optoacoustic imaging. By employing the hemoglobin as light absorber, optoacoustics can provide detailed images of both small (diameter < 150–200 µm) and larger vessels in microscopic, mesoscopic and macroscopic applications. For example in (14), the authors employ optoacoustic microscopy (OAM) to achieve functional imaging of vascular dynamics in the finger, extracting invaluable information about heart rate, blood oxygenation and perfusion. In another work, raster-scan optoacoustic mesoscopy (RSOM) demonstrated capability to assess vascular anomalies, such as capillary malformations (CMs) and infantile hemangiomas (IHs) before and after the application of a corresponding treatment (15). Regarding larger arteries, optoacoustics has been also employed in imaging, for example, the radial artery morphology and oxygenation (16, 17). Nevertheless, arteries such as the radial are quite superficial (depth of 2–3 mm under the skin), a feature that facilitates their imaging using optoacoustics, a technology affected by light attenuation with increasing depth. On the contrary, in the case of the carotid artery, *in vivo* imaging using optoacoustics remains challenging.

In this work, we explore the applications and potentials of both native (label-free) and contrast-enhanced optoacoustic imaging of carotid atherosclerosis (Table 1). As observed, such applications refer mainly to inflammatory and lipid-metabolism processes because of the unique capability of optoacoustics to images relevant biomarkers (e.g., hemoglobin, lipids) based on their characteristic light absorption properties (11, 12, 18, 19, 31). Moreover, even if we focus on clinical applications, we also describe preclinical studies with high translational interest. Finally, we provide insights into the future of the technology as a useful tool for the vascular specialist dealing with carotid atherosclerosis.

**Table 1:** Overview of the studies employing optoacoustics for carotid artery imaging.

Type of study (*N*)	Spatial resolution	Real-time imaging (scale)	Field of view	Effective imaging depth	Illumination wavelengths	Reconstruction method	Spectral analysis (method)	Ultrasound detection	Other imaging modalities	Laser	Histological validation	Possible biomarkers resolved	Chromophore, contrast agent	Energy per pulse
Preclinical ex vivo (excised plaques)														
Seeger, Karlas et al. 2016 (*N* = 1pl) (20)	∼1 *μ*m lateral	No (min)	1 × 1 cm (low-resolution), 630 × 630 µm (high-resolution)	∼300 *μ*m	515 nm	NR	No	One 100 MHz transducer	SHG, THG, TPEF	Diode-pumped solid-state	Yes	IPH, angiogenesis	Hemoglobin	570 *μ*J
Visscher, Pleitez et al. 2022 (*N* = 3pl) (24)	25 µm, 2.5 µm or 5 µm	No (min)	0.5 × 0.5 cm	NR	2941–2780 cm^−1^ and 1739–909 cm^−1^, steps of 2–4 cm^−1^	NR	No	One 20 MHz transducer	No	Quantum cascade	Yes	Inflammation, necrosis	Lipids, proteins, carbohydrates	NR
Arabul et al. 2017 (*N* = 7pl) (23)	0.28 mm axial and 0.5 mm lateral	No (min)	∼1.5 × 1.5 cm	∼2 cm	808 nm	Delay-and-sum	No	Linear array transducer, 7.5 MHz	Ultrasound	Diode	Yes	IPH, angiogenesis	Hemoglobin	1 mJ
Arabul et al. 2019 (*N* = 6pl) (33)	0.28 mm axial and 0.5 mm lateral	No (min)	∼1.5 × 1.5 cm	∼2 cm	808 nm, 915 nm, 940 nm, 980 nm	Delay-and-sum	Yes (Blind source NN ICA)	Linear array transducer, 7.5 MHz	Ultrasound	Diode	Yes	Recent and old IPH, lipid core	Hemoglobin, cholesterol	1 mJ
Johnson et al. 2018 (*N* = 1pl) (26)	NR	No (min)	∼1.5 × 1.5 cm	∼1 cm	Source: 680 nm, detection: 1450 nm	Time reversal, reverse-time migration	No	Laser-Doppler vibrometer, detection laser 1450 nm	Computed tomography	Tunable OPO	Yes	Positive artery wall remodeling, calcification	Calcium	20 mJ/cm^2^ (source), 40 mJ/cm^2^ (detection)
Kruizinga et al. 2014 (*N* = 1pl) (22)	NR	NR	∼2 × 2.5 cm	2–3 cm	25 wavelengths, 1130 : 5 : 1250 nm	NR	Yes (Least squares)	Linear array, 8 MHz	Ultrasound	Tunable pulsed	Yes	Lipid core	Lipids, collagen	2.5 mJ
Razansky et al. 2012 (*N* = 5pl) (25)	< 200 µm	No (min)	∼1.5 × 1.5 cm	1–1.5 cm	635 nm, 675 nm	Two-dimensional filtered back-projection	No	Wideband piezoelectric PZT transducer, 3.5 MHz	Fluorescence	Tunable MOPO	Yes	Inflammation	MMPSense™ 680	2 mJ/cm^2^
Cano et al. 2023 (N = 9pl) (35)	NR	No (min)	∼1 × 1 cm	∼1 cm	580 nm, 600 nm, 720 nm, 900 nm, 1100 nm, 1200 nm	Delay-and-sum beamforming	Yes (Blind unmixing)	Linear array of 128 transducers, 7.6 MHz	Ultrasound	Tunable OPO	Yes	IPH, lipid core, mechanical stability	Lipids, collagen, methemoglobin	NR
Preclinical *in vivo* (animal models)														
Li et al. 2017 (*N* = 20 mice: 15d/5c) (38)	200 µm	Yes	∼2 × 2cm	∼2 cm	800 nm	NR	No	Array of 128 transducers, 5 MHz	No	Tunable pulsed Nd:YAG	Yes	Thrombosis	Hemoglobin	NR
Xie et al. 2020 (*N* = 15 mice: 10d/5c) (39)	NR	No (min)	14 × 10 mm	2–3 mm	1064 nm	NR	No	One 10 MHz transducer	Ultrasound	OPO	Yes	Inflammation	PBD-CD36	12 mJ/cm^2^
Clinical (patients with carotid artery disease)														
Karlas, Kallmayer et al. 2021 (*N* = 10p, 10h, 2pl) (32)	200–300 µm	Yes	4 × 4 cm	2–4 cm	28 wavelengths 700 : 10 : 970 nm	Model-based	Yes (Linear unmixing)	Array of 256 transducers, 4 MHz	Ultrasound	Tunable pulsed laser	Yes	Inflammation, IPH, angiogenesis, lipid core	Hemoglobin, lipids	<15 mJ/cm^2^
Yang et al. 2020 (*N* = 10p, 3h) (45)	200–300 µm	Yes	4 × 4 cm	2–4 cm	800 nm, 850 nm, 930 nm	Least-squares and prior-integrated	No	Array of 256 transducers, 4 MHz	Ultrasound	Tunable pulsed laser	No	Inflammation, IPH, angiogenesis, lipid core	Hemoglobin, lipids	25 mJ
Muller et al. 2021 (*N* = 16p) (40)	Lateral: 0.4–0.6 mm, axial: 0.28 mm	Yes	∼1 × 1 cm	∼8 mm	808 nm, 940 nm	Fourier reconstruction algorithm	No	Linear array with 64 elements, 7 MHz	Ultrasound	Two laser diodes	Yes	IPH, lipid core	Hemoglobin, lipids	1 mJ/cm^2^
Steinkamp et al. 2021 (*N* = 4p, 5h) (46)	∼180 *µ*m	Yes	4 × 4 cm	4 cm	700 nm, 730 nm, 760 nm, 780 nm, 800 nm, 850 nm	Back-projection	Yes (NR)	Array of 256 transducers, 4 MHz	Ultrasound, fluorescence	Pulsed Nd: YAG	Yes	Neovascularization	Hemoglobin, bevacizumab-800CW	25 mJ

NR, not reported; ROI, region of interest; IPH, intraplaque hemorrhage; SHG, second harmonic generation; THG, third harmonic generation; TPEF, two photon fluorescence; MiROM, mid-infrared optoacoustic microscopy; MMP, matrix metalloproteinase, OPO, optical parametric oscillator; pl, plaque; d, diseased; c, controls; p, patients; h, healthy volunteers.

## Excised plaque imaging

**Figure 3:**
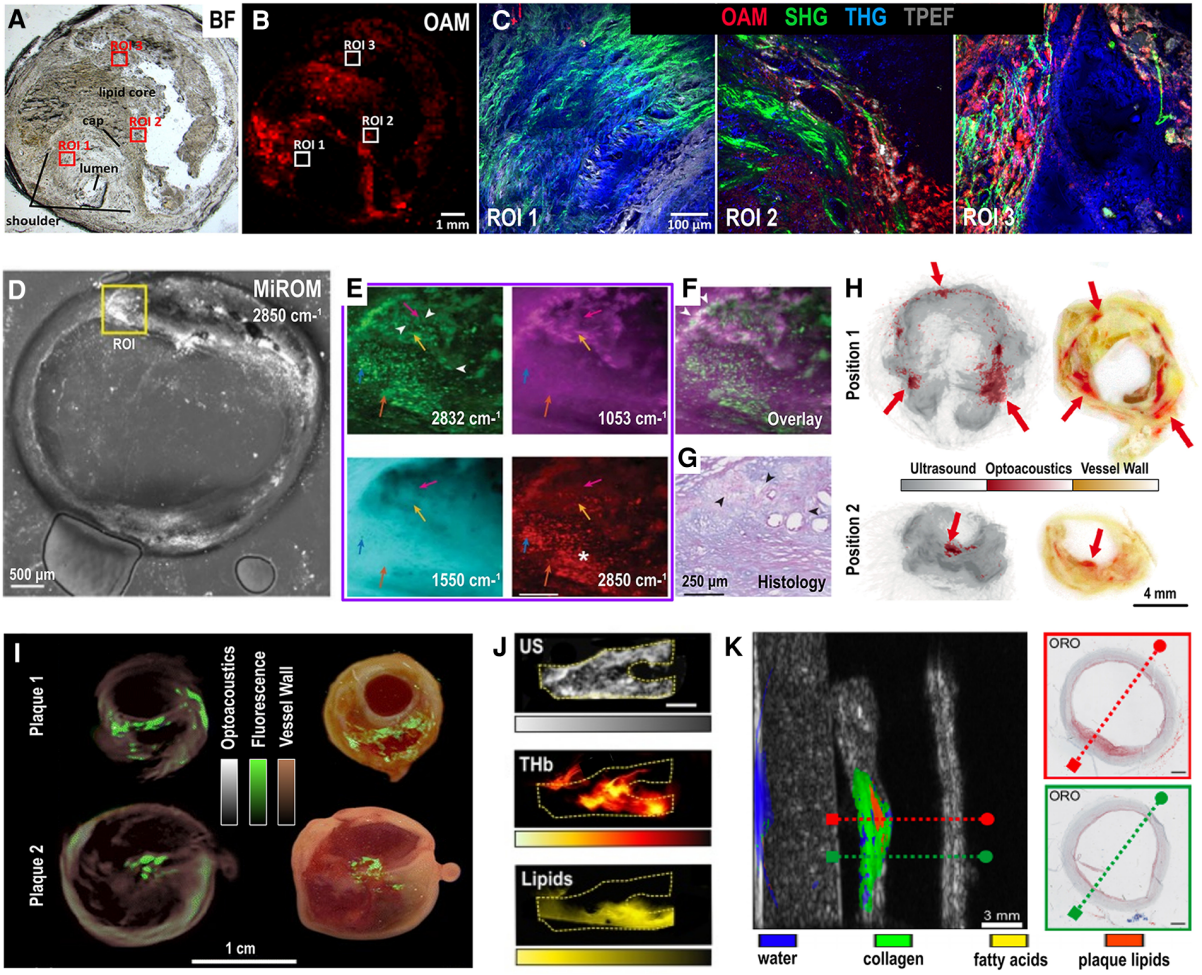
*Ex vivo* optoacoustic imaging of human carotid plaques. (**A**) Bright-field (BF) visualization of an unstained excised carotid plaque with three regions of interest (ROIs) corresponding to the shoulder (ROI 1), cap (ROI 2) and the lipid core (ROI 3). (**B**) Sample overview with optoacoustic microscopy (OAM) showing intraplaque red blood cells (RBCs). (**C**) Left: Multimodal microscopy of the unstained plaque over the ROI 1,2 and 3. Colormap overlay: red-OAM-RBCs, green-second harmonic generation-collagen, blue-third harmonic generation-tissue morphology, grayscale-two-photon fluorescence-elastin. (**A**–**C**) adapted from (20). (**D**) Mid-infrared optoacoustic microscopy (MiROM) image of excised carotid plaque at 2,850 cm^−1^. (**E**) Images at 2,832 cm^−1^ (up-left), 1,053 cm^−1^ (up-right), 1,550 cm^−1^ (down-left, protein) and 2,850 cm^−1^ (down-right, lipids) of the ROI shown in (**D**), arrowheads: cholesterol crystals, *: lipid droplets. (**F**) Overlay of 1,053 cm^−1^ (magenta) and 2,832 cm^−1^ (green), arrowheads: overlapping signals. (**G**) Histology with Periodic acid-Schiff (PAS) and Alcian Blue (AB) stainings, showing carbohydrate macromolecules like glycogen and glycolipids (PAS, purple) and acidic mucosubstances (AB, blue), arrowheads: cholesterol crystal clefts. (**D–G**) adapted from (24). (**H**) Two (up and down) cross-sections of an excised human carotid plaque: Left - overlay of optoacoustic (red, 808 nm) and ultrasound (grayscale) images, right—corresponding histology sections without staining, red arrows: intraplaque blood embeddings. Adapted from (23). (**I**) Optoacoustic imaging (635 nm, grayscale) of two carotid plaque samples along with fluorescence overlay (green colormap) of matrix metalloproteinase activity (680 nm) and corresponding color plaque images (right column). Adapted from (25). (**J**) Up: Ultrasound (US) imaging of an excised human carotid plaque, middle: Spectrally unmixed optoacoustic image of the same plaque showing the total hemoglobin (THb) distribution, down: Lipid-specific spectrally unmixed optoacoustic image of the same plaque. Adapted from (32). Scale bar: 4 mm. (**K**) Left: Hybrid optoacoustic-ultrasound imaging of a plaque with the water (blue), collagen (green), fatty acids (yellow) and plaque lipids (orange) distributions. Right (up-down): Two histologic images with Oil Red O staining (ORO, lipids) corresponding to the planes also indicated with red and green dashed lines in the corresponding left plaque visualization. Adapted from (22).

Several microscopy studies have been conducted to study excised carotid samples seeking novel insights into disease pathophysiology without the need for staining (Figure 3). In (20), a multimodal microscope combining optoacoustic microscopy (OAM) along with second (SHG) and third (THG) harmonic generation and two-photon excitation microscopy (TPEF) has been employed to simultaneously image several features of a human carotid plaque with clinical significance (Figures 3A–C). More specifically, OAM provided intraplaque images of blood by exciting the sample at 515 nm (energy per pulse: 570 uJ), SHG co-registered images of collagens (type I and II), THG of tissue morphology and TPEF of elastin and lipids. Presence or degradation of the abovementioned features (e.g., presence of blood which indicates neoangiogenesis and intraplaque hemorrhage, degradation of collagens within the fibrous cap and presence of a large lipid core) are indicative of plaque instability, based on histological analyses (21). The system provided multimodal imaging of the plaque with a lateral resolution of ∼1 µm for all modalities, showcasing the capability of OAM to be combined with other staining-free modalities to provide complementary information about critical biological processes.

In (24), mid-infrared optoacoustic microscopy (MiROM), a newly developed technique able to provide microscopic imaging of several molecules within a sample (8, 28), was employed to explore the molecular composition of three human carotid plaques. By employing a tunable pulsed quantum cascade laser (QCL) operating at the range of 2,932–2,770 cm^−1^ and 1,739–900 cm^−1^, MiROM provided maps of different biomolecules within different plaque regions at a resolution of down to 2.5 µm. In specific, lipid, protein and carbohydrate spatial maps were revealed mainly at the 2,850, 1,550 and 1,053 cm^−1^, correspondingly (Figures 3D–G). The study demonstrates how optoacoustic microscopy can provide biochemical characterization of complex excised carotid plaques in a label-free mode.

Human plaques have been also imaged as intact samples after their excision via carotid endarterectomy (CEA). In (23), a custom-made system was used to image intraplaque hemorrhage and neoangiogenesis, two features corresponding to blood content of the plaque. Seven plaques were excised from patients with symptomatic carotid stenosis and scanned with the hybrid optoacoustic-ultrasound (US) probe. By illuminating at 808 nm (pulse energy: 1 mJ), rotating for 360° and employing US to capture morphology, the system provided 3D visualizations of plaque blood content and morphology at a 0.5 mm lateral and 0.28 mm axial resolution without the need for extra contrast agents. However, to reveal different components or chromophores within the plaque, and achieve, thus, a more complete plaque characterization, illumination at many different wavelengths or else multispectral optoacoustic imaging should be conducted. For example, in (33) human carotid plaques were excited at four NIR wavelengths: 808, 915, 940 and 980 nm using the same experimental setup as in (23). Via a blind spectral unmixing step based on independent component analysis (ICA), the authors were able to discriminate between fresh and old intraplaque hemorrhages. The findings, which were histologically confirmed, demonstrate the capability of optoacoustic imaging to provide assessment of complex tissue compositions, such as the carotid plaque.

But apart from label-free imaging, optoacoustic systems can also detect molecule-specific dyes targeting specific intraplaque processes, such as inflammation. For example, in (25) plaques excised from five symptomatic patients were first incubated for 60 min in a suspension of an inflammation-sensitive probe (MMPSense™ 680) in 37°C and then imaged with a preclinical MSOT system with a resolution of 200 µm (Figure 3I). The plaques were illuminated at the 635–675 nm range. MSOT images acquired at 635 nm served as morphologic plaque maps, while the ones at 675 nm represented the co-registered distribution of the inflammation-sensitive probe, which is characterized high excitation efficiency around the 680 nm. In fact, the probe indicated the intraplaque presence of matrix metalloproteinases (MMPs), a group of enzymes, which indicate inflammatory activity and plaque instability (34). Considering that the used probe exhibits also fluorescence properties, the inflammatory activation in the excised carotid samples, was validated using a fluorescence imaging system with a charged coupled device (CCD) camera and illumination at 675 nm.

The hemoglobin and lipid content of excised human carotid plaques were also imaged in (32) using MSOT. By illuminating tissue at the near-infrared range of 700–970 nm, high resolution (200–300 µm) maps of intraplaque hemoglobin and lipids were produced, as shown in Figure 3J which demonstrates the total hemoglobin (THb), lipids and US images of a plaque. A more detailed description of the study, which also includes *in vivo* imaging and characterization of human plaques is provided in the “Clinical studies” section.

Along the same lines, the composition of excised human carotid samples was also assessed in (35). More specifically, both a plaque mimicking phantom and nine *ex vivo* carotid endarterectomy samples were measured with a custom-made optoacoustic imaging setup operating at 500–1,300 nm. Acquired optoacoustic images were spectrally unmixed via a blind unmixing approach to calculate methemoglobin, lipids and collagen and assess, thus, the intraplaque hemorrhage, lipid content and mechanical stability of the plaque. The results were also validated by means of histology.

The presence of calcification is also a pathologoanatomic feature associated with carotid plaque vulnerability (36). The authors in (26), combined an all-optical extravascular laser-ultrasound system (LUS) in combination with OA to image a carotid artery excised collected at autopsy. The system employed two different lasers, a source and a detection one. In fact, the source laser wavelength could be tuned in order to switch between the LUS and OA operation modes and image the arterial wall structure and calcification (LUS at 1,450 nm) or the optical properties/hemoglobin content (OA at 680 nm) of the plaque. The detection laser is embedded into a laser-Doppler vibrometer (LDV) and enabled the detection of the generated ultrasound waves. While the OA component of the system employed laser fluence of 20 mJ/cm^2^, or else the permissible energy limit for human use (27), the LUS employed 40 mJ/cm^2^ laser fluence. The results are validated with CT and histology.

In another study, optoacoustic spectroscopic imaging of an anatomy-mimicking phantom containing a cadaveric common carotid artery (CCA) with atherosclerosis (Figure 3K), provided lipid-specific information within the collagen structure of the arterial wall (22). The sample was illuminated at 1,130–1,250 nm using a custom-made optoacoustic system based on an optical fiber and a separate linear ultrasound detector to simulate an internal (pharynx) illumination and external (cervical skin) detection principle-of-operation. The output fluence of the probe was ∼12.7 mJ/cm^2^ (2.5 mJ for a spot of ∼5 mm in diameter). The presented method: (i) could spectrally differentiate among water, collagen, fatty acids and plaque lipids, a step also validated by histology and (ii) demonstrated feasibility of a different configuration than that of external (skin) illumination and detection which is usually used in current optoacoustic systems.

## Small animal models

*In vivo* imaging of the carotid artery has been demonstrated in animal models. More specifically, in (29) the carotid arteries of mice were imaged by illuminating the cervical region at 750 nm by means of a preclinical system operating at the NIR (700–900 nm). The fluence on the mouse skin was estimated to be 19 mJ/cm^2^ at the 740 nm, just below the limit for human use (20 mJ/cm^2^), as described above. The system allowed for high-resolution (∼150 µm) *in vivo* mouse imaging and was equipped with a linear stage to enable imaging at multiple transverse plains while moving the mouse through a ring-shaped transducer array (37). The study demonstrated the capability of MSOT to resolve vessels in the order of hundreds of microns, which is sufficient for imaging the mouse carotid artery.

**Figure 4:**
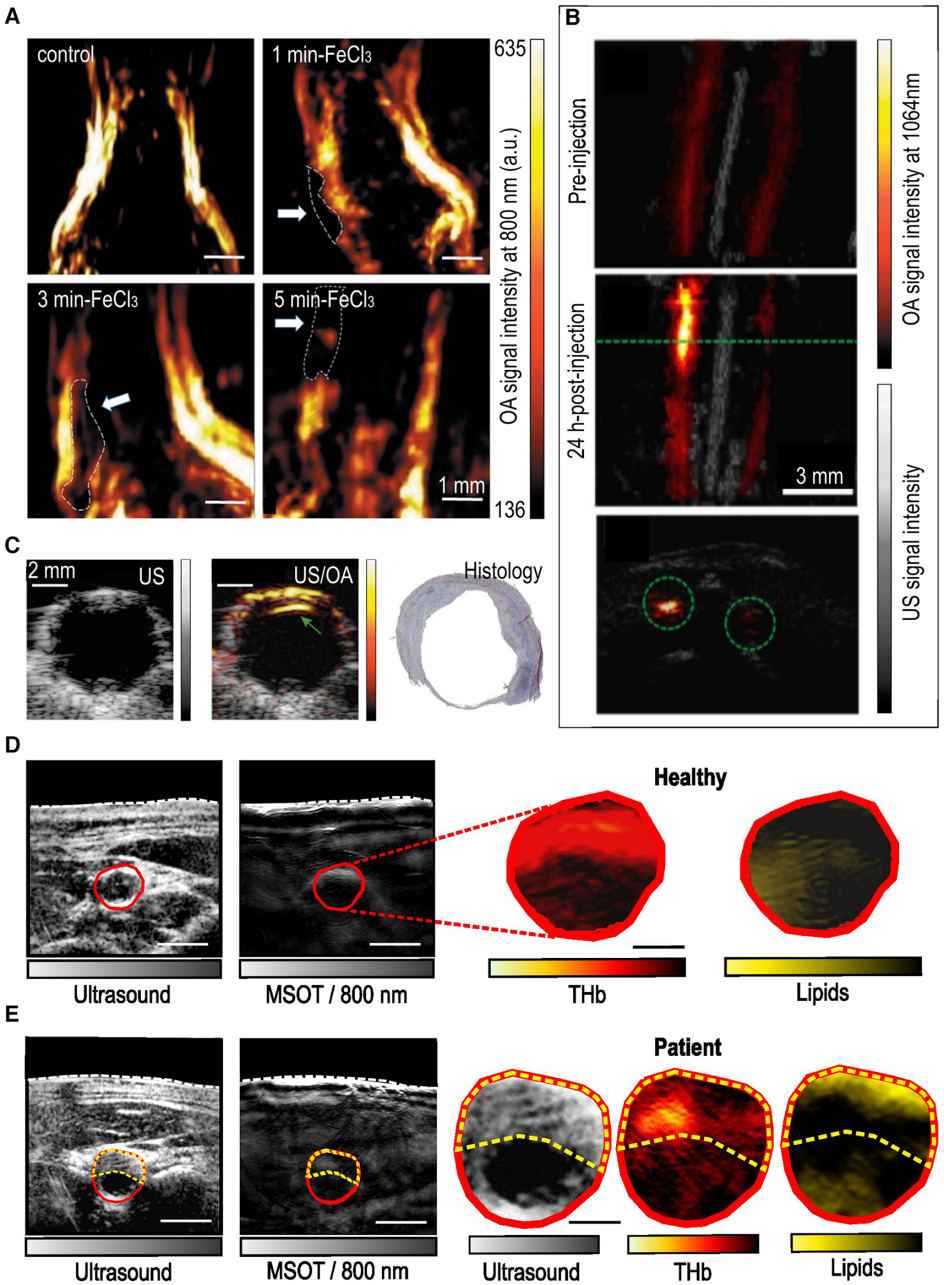
*In vivo* imaging of carotid lesions in animal models and humans. (**A**) Optoacoustic imaging (800 nm) of mouse carotid arteries in a carotid thrombosis model. Up-left: control, up-right: mouse carotid treated with FeCl3 for 3 min, down-left: for 3 min and down-right: for 5 min. The white arrows indicate the locations of the thrombi in the FeCl3-treated mice. Adapted with permission from (38). (**B**) Optoacoustic (1,064 nm)—ultrasound overlays of the carotid arteries/plaques of a mouse before (up) and 24 h after (middle and down) the intravenous injection of a molecular probe (PBD-CD36 NP) targeting inflammation. The green dashed line in the middle corresponds to the same plane of the lower image. Adapted with permission from (39). (**C**) *In vivo* intraoperative (during carotid endarterectomy) imaging (left-ultrasound, middle-overlay of optoacoustic and ultrasound images) of a human carotid plaque. Gray colorbar: ultrasound signal intensity, yellow-red colorbar: optoacoustic signal at 808 nm. Right: Histology analysis with Masson's trichrome staining of the sample. Adapted with permission from (40). (**D**) Non-invasive imaging of a healthy human common carotid artery (CCA, indicated with a red contour). Left: Ultrasound and 800nm-optoacoustic image, right: unmixed optoacoustic image of the carotid artery for hemoglobin (total, THb) and lipids. (**E**) Non-invasive imaging of a CCA with a plaque (yellow dashed contour). Left two images: same as in (**D**). Right three images: Zoom-in of the CCA cross-section in ultrasound and unmixed optoacoustic images for THb and lipids. Scale bars: 2.5 mm. Adapted with permission from (32).

In (38), OA was employed to detect thrombosis of the carotid artery in a corresponding animal model (Figure 4A). Carotid artery thrombosis was induced by an iatrogenic chemical injury via the application of a filter paper soaked with 20% FeCl_3_ around the vessel wall after the surgical exposure of the carotid artery. In total, 20 mice were scanned after being categorized into 4 groups: a control group and 3 groups with different durations of FeCl_3_ application, namely 1, 3 and 5 min. The carotid arteries were illuminated at 800 nm: the isosbestic absorption point of Hb. OA was able to detect the presence of thrombi within the carotid artery via a considerable decrease in the lumen region and the measured intravascular signal intensities: a decrease reflecting reduction in the intravascular Hb-content or else increase in the thrombus mass. The reduction in the measured optoacoustic signals followed the thrombosis severity, as induced by increasing FeCl_3_-exposure times. The presence of thrombi in the carotid artery was also validated via ultrasound (30 MHz central frequency).

OA has been also employed in imaging carotid plaque inflammation in mice (39). Mice were injected with an anti-CD36 decorated semiconducting polymer NP (PBD-CD36 NP), which is characterized by an absorption peak at 1,064 nm (Figure 4B). Optoacoustic imaging was conducted using a custom-made system also equipped with ultrasound (41). Mice were illuminated at 1,064 nm with a fluence of 12 mJ/cm^2^. The increased optoacoustic signal measured reflected the expression of CD36(+) in the plaques, as validated by immunochemistry, demonstrating the capability of optoacoustics to provide *in vivo* image the intraplaque inflammation in carotid atherosclerosis.

## Clinical studies

Even if imaging of excised carotid plaques or animal models is of great interest for basic research and deeper understanding of disease pathophysiology, *in vivo* imaging is the absolute goal of every imaging modality. The capability of MSOT to visualize the common carotid artery, as well as the carotid bifurcation, despite the limited penetration depth (2–4 cm), has been already demonstrated (30, 42). Moreover, another implementation of hand-held optoacoustic imaging (43, 44) has been used to image the carotid plaques intraoperatively (Figure 4C) by applying the probe directly on the carotid artery during the planned surgical excision of the plaque via CEA (40). This way, the authors avoided the effect of superficial soft tissue layers, such as the skin, subcutaneous fat, veins and muscles that could cause strong light attenuation or spectral coloring, phenomena that can decrease the imaging depth and contaminate the results (7). The system was equipped with two laser diodes at 808 and 940 nm and a linear US array with 64 elements with a central frequency of 7 MHz offering simultaneous and co-registered OA/US imaging with a very low per-pulse energy of 1 mJ/cm^2^. The 808 nm were used to image the total hemoglobin, a marker of intraplaque blood content/hemorrhage and, thus, instability. The 940 nm corresponded to lipids, although the authors focused solely on the intraplaque hemorrhage. In total plaques from *N* = 16 patients were scanned with each plaque examined over 3–5 positions. Results showed that, by applying the abovementioned scheme, intraplaque hemorrhage could be detected in human plaques *in vivo*, as validated by ex vivo OA/US scans and histology. Finally, the hybrid nature (OA/US) of the employed system allowed for the development of a motion correction method (motion correction averaging, MCA) relying on US-based speckle tracking. The MCA step increased the optoacoustic signal-to-noise-ratio (SNR) by 5 dB.

Going one step further, the authors in (32) were able to visualize not only the plaques in patients with previously diagnosed carotid atherosclerosis but also to describe the intraplaque lipid and blood content using clinical MSOT (Figures 4D,E). More specifically, five asymptomatic patients with carotid stenosis and five healthy volunteers were scanned with MSOT over the carotid artery. The illumination scheme included 28 wavelengths (700–970 nm at 10 nm-steps) offering rich spectral information which enabled the clear MSOT-differentiation between healthy and diseased carotid arteries and between the lumen and plaque regions in the patient group. The above-described differentiations were based on the characteristic spectra of lipids and hemoglobin detected by MSOT within the corresponding regions of interest (ROIs). The results were validated by scanning the excised plaques with the same MSOT system, as well as by histology.

In another study (45), the co-registered structural US data, which were recorded along with MSOT, were used to improve the optoacoustic images by reducing limited-view artifacts and increasing contrast. More specifically, the carotid artery lumen and plaque areas were segmented in the US images from three healthy volunteers and five patients with asymptomatic carotid atherosclerosis. Then, the segmentation masks were used as US-based priors to improve the reconstruction of several optoacoustic frames, including the frames acquired at the 930 nm, where the lipids absorb the most at the NIR of 700–1,000 nm (45). The results showed that the prior-integrated reconstruction rendered the differentiation of the two groups (healthy vs. patients) statistically significant in contrast to the standard reconstruction regime, when based on the optoacoustic signal intensities recorded at 930 nm.

Finally, in (46) the authors employed MSOT in an attempt to image the neoangiogenesis in the carotid plaques of patients with symptomatic stenosis after administering intravenously a low dose of bevacizumab-800CW (4.5 mg) three days before the planned carotid endarterectomy. Bevacizumab-800CW is a near-infrared tracer which targets vascular endothelial growth factor-A (VEGF-A), a molecule overexpressed in the presence of intraplaque neovascularization: an indicator of plaque vulnerability. MSOT was performed in four patients and could not detect bevacizumab-800CW within the plaques *in vivo*, either because the injected dose was too low or the tracer selected was not optimal for optoacoustic signal generator, as hypothesized by the authors. The hybrid MSOT/US system (MSOT Acuity Echo prototype; iThera Medical GmbH, Oberschleißheim, Germany) employed a 25 Hz pulsed Nd: YAG laser with a laser output of 25 mJ per pulse and a resolution of 180 µm. The cervical region of the patients was illuminated with six wavelengths (700, 730, 760, 780, 800 and 850 nm). Even if MSOT was not able to *in vivo* detect the bevacizumab-800CW, the tracer was detected within the excised plaque sample using electrophoresis (sodium dodecyl sulfate–polyacrylamide gel electrophoresis, SDS-Page).

## Conclusions and future steps

Optoacoustics comes with several characteristics that render it useful in imaging the carotid artery and pursuing the vulnerable plaque. First, by employing the same phenomenon in different system configurations, optoacoustics can provide a multiscale, but always high resolution, assessment of the carotid plaque along different platforms, starting from the macroscopic *in vivo* assessment in humans to imaging in small animals or even meso- and microscopic imaging of excised specimens. Second, by enabling the acquisition of tissue responses at different illumination wavelengths, or else of multispectral data (e.g., with MSOT), optoacoustics offer the unique opportunity to resolve the intraplaque distribution either of endogenous molecules, such as hemoglobin and lipids, or of injected dyes (e.g., indocyanine green, ICG) based solely on their characteristic absorption spectrum. For example, MSOT is capable of investigating different aspects of disease pathophysiology by imaging both endogenous and exogenous contrasts simultaneously within the same tissue area (47).

Third, optoacoustics provides up to six-dimensional (6D) imaging depending on the system configuration: three-dimensional space (3D), time (4D), spectrum (5D) and frequency (6D). With this rich amount of information many different aspects of the disease can be explored. Fourth, it is a highly portable and safe technology which may be well combined with US in hybrid configurations to provide co-registered images of anatomy, function and metabolism in real time.

Of course, optoacoustics has also disadvantages that might hinder its use in imaging the carotid artery, mainly in clinical studies. In fact, the penetration depth of current clinical systems is approximately 2–4 cm based upon tissue type, when operating below the highest energy allowed for human use. Thus, optoacoustic imaging of the carotid artery might be extremely challenging in subjects where the carotid artery lies deep within the cervical soft tissues (e.g., obese patients). Furthermore, even at these depths, the spectral coloring effect (the different interaction between each laser wavelength/pulse and tissues with depth) leads to significant distortion of the measured spectrum at higher depths, decreasing the accuracy of the spectral unmixing and, thus, of quantifying chromophores such as hemoglobin and lipids for each image pixel. Finally, optoacoustic spectral unmixing, is vulnerable to motion artifacts either due to motion of the probe on the imaged tissue or even motion of the carotid artery wall due to pulsation. Thus, accurate quantification of chromophores in deep tissue areas (e.g., lipids in the carotid plaque) might be a challenging task.

However, several solutions have been suggested to address the limitations described above. For example, the development and application of tailored motion correction algorithms could help improving the accuracy of current spectral unmixing methods (48). Also, several innovative spectral unmixing techniques have been developed to address the spectral coloring effect, or else the distortion of the measured absorption spectrum with depth because of the different attenuation of each wavelength/laser pulse when propagating through tissues (49). For example, blind unmixing, statistical or deep learning-based techniques offer the opportunity to overcome the effect of light-tissue interaction phenomena (e.g., absorption, scattering) (35, 50, 51).

Of course, as inflammation plays an important role in atherosclerosis it might be challenging to differentiate atherosclerosis from other inflammatory conditions, such as vasculitis, by means of imaging. The differentiation of these two inflammatory conditions is critical in order to choose and follow the right therapeutic strategy. MRI studies showed that vasculitis is characterized by a concentric wall thickening and intrawall contrast uptake while atherosclerotic lesions by clear eccentric morphology and no contrast uptake (52, 53). Optoacoustics, with its unique capability to image both morphology and disease activity, could allow to differentiate between such pathologoanatomic features based on hemoglobin and lipid contrast.

Having this entire in mind, optoacoustics demonstrates great potential for imaging carotid atherosclerosis not only in excised plaques or small animal models, but also in humans. Further large-scale and longitudinal studies are needed to validate the accuracy of the technology in the detection of different pathophysiological processes connected to plaque vulnerability. Such studies should focus on several unanswered questions, such as the mechanisms governing the recurrence of carotid artery stenosis in some patients, the plaque characteristics that could be associated with plaque rupture or the effect of novel pharmacological therapies on plaque evolution and stabilization.
